# Delphi panel consensus on genetic testing for prostate cancer in Australia: Whom to test and how?

**DOI:** 10.1007/s10689-025-00528-x

**Published:** 2026-01-24

**Authors:** Kim Edmunds, Shiksha Arora, Sri Teppala, Paul Scuffham, David Fairbairn, Matthew J. Roberts, Lisa Horvath, David P. Smith, Haitham Tuffaha

**Affiliations:** 1 Centre for the Business and Economics of Health, University of Queensland, Brisbane, Australia; 2 Centre for Applied Health Economics, School of Medicine & Dentistry, Griffith University, Gold Coast, Australia; 3 Pathology Queensland, The Royal Brisbane Women’s Hospital, Brisbane, Australia; 4 UQ Centre for Clinical Research, University of Queensland, Brisbane, Australia; 5 Department of Urology, Royal Brisbane and Women’s Hospital, Brisbane, Australia; 6 Medical Oncology, Chris O’Brien Lifehouse, Camperdown, NSW Australia; 7 Clinical Prostate Cancer Group, Garvan Institute of Medical Research, Darlinghurst, NSW Australia; 8 Faculty of Medicine and Health, University of Sydney, Camperdown, NSW Australia; 9 The University of Sydney, A Joint Venture with Cancer Council NSW, The Daffodil Centre, Sydney, Australia

**Keywords:** Genetic testing, Genetic counselling, Prostate cancer, Delphi panel study

## Abstract

The purpose of this study was to estimate the consensus of Australian Health Professionals and/or Researchers (HP/Rs) and Consumers (patients/family/carers) (Cs) on international genetic testing recommendations. Modified Delphi Panel study with seven domains of interest. Fifty-five statements were devised for an online survey administered in REDCap. HP/Rs and Cs were recruited from professional networks and associations for participation in the study. Statements were rated using a Likert scale and analysed using descriptive statistics. Free text comments were allowed within each domain and analysed using thematic analysis. Thirty-six HP/Rs and 27 Cs participated. There was consensus on testing men with a family history of a high-risk hereditary gene; men with PCa and a family history of Hereditary Breast and Ovarian Cancer (HBOC) syndrome or Lynch syndrome; and men with metastatic PCa. There was consensus on testing *BRCA2, BRCA1* and *DNA MMR* genes for men with metastatic PCa. Thematic analysis of HP/R comments revealed three main topics: the lack of information to make a decision, insufficient knowledge of genetic testing, and capacity to provide genetic testing and counselling. This is the first Australian study on genetic testing recommendations in PCa to inform who should be tested and how. Our study showed apparent deficits in knowledge and implementation, exacerbated by workforce issues around the provision of genetic counselling and testing. Future work should focus on evaluating these recommendations for implementation in Australian practice.

**Supplementary Information:**

The online version contains supplementary material available at 10.1007/s10689-025-00528-x.

## Introduction

Prostate cancer (PCa) is the most common cancer in males in Australia (~ 26,400 cases diagnosed in 2024) [1]. The estimated annual cost of PCa treatment to Australia (2022–2023) is $1.9 billion, the highest healthcare spending of all cancers [2]. PCa can be heritable (5–15%) [3], with mutations occurring in homologous recombination repair (HRR) genes, such as *BRCA2, BRCA1, ATM, CHEK2,* and mismatch repair (MMR) genes (*MLH1, MSH2, PMS2* and *MSH6*). The presence of an inherited pathogenic variant in one of these genes (e.g. *BRCA1* and *BRCA2*), is associated with early onset, aggressive disease and less favorable outcomes [3–7].

Germline testing and genetic counselling have implications for the prevention and treatment of PCa and the potential to improve the efficiency of healthcare and mitigate some of the costs [3, 8]. There is need for a paradigm shift in current PCa treatment practice towards precision care that includes targeted treatments for men with PCa, so potentially toxic treatments are reserved for those who stand to benefit most (e.g., predicting response to specific therapies such as poly-ADP-ribose polymerase (PARP) inhibitors for men with advanced PCa). Genetic testing also enables risk-based early detection in first degree family members by identifying those who share the same variants, so that curative treatment can be more effective (e.g., women who have the BRCA mutation at high risk of breast or ovarian cancer). Before and after testing, genetic counselling is essential in helping patients and family members to better understand and navigate the implications of genetic testing. Counsellors provide psychosocial support and facilitate informed decision making around testing results and the implications for family planning and cascade testing. Guidelines for genetic testing in PCa are an important component of a paradigm shift towards precision care.

International guidelines and consensus statements for genetic testing in PCa recommend genetic testing for men with PCa and for individuals at high risk of developing PCa (e.g., due to family history or ancestry) [9]. However, there is considerable variation amongst existing guidelines [9]. Importantly, while recommendations for genetic testing in Australia are provided by eviQ, there is no national guideline in Australia for genetic testing in PCa, and the access to genetic testing in PCa via the Medicare Benefits Scheme is limited to PCa patients with castration-resistant disease who are candidates for targeted treatment (Items 73,303 and 73,304). To enhance patient access to genetic testing for PCa, there is a need to identify genetic testing strategies (i.e., who should be tested, when and how) and evaluate those strategies for effectiveness and value for money. This Delphi study aims to estimate the consensus of Australian Health Professionals and/or Researchers (HP/Rs) and Consumers (patients/family/carers) (Cs) on international genetic testing recommendations.

## Subjects and methods

### Study design

A Delphi study uses a scientific method to generate insights and expert opinion from a group of anonymous stakeholders, particularly where there is diversity of views or limited availability of information [10]. The Delphi technique typically consists of at least two rounds and can include a final discussion round where practicable. The aggregated group opinion is provided to participants after each round. The purpose of this approach is to contribute to convergence (or divergence) of opinions to produce more accurate results than traditional opinion polling approaches [10].

**Fig. 1:**
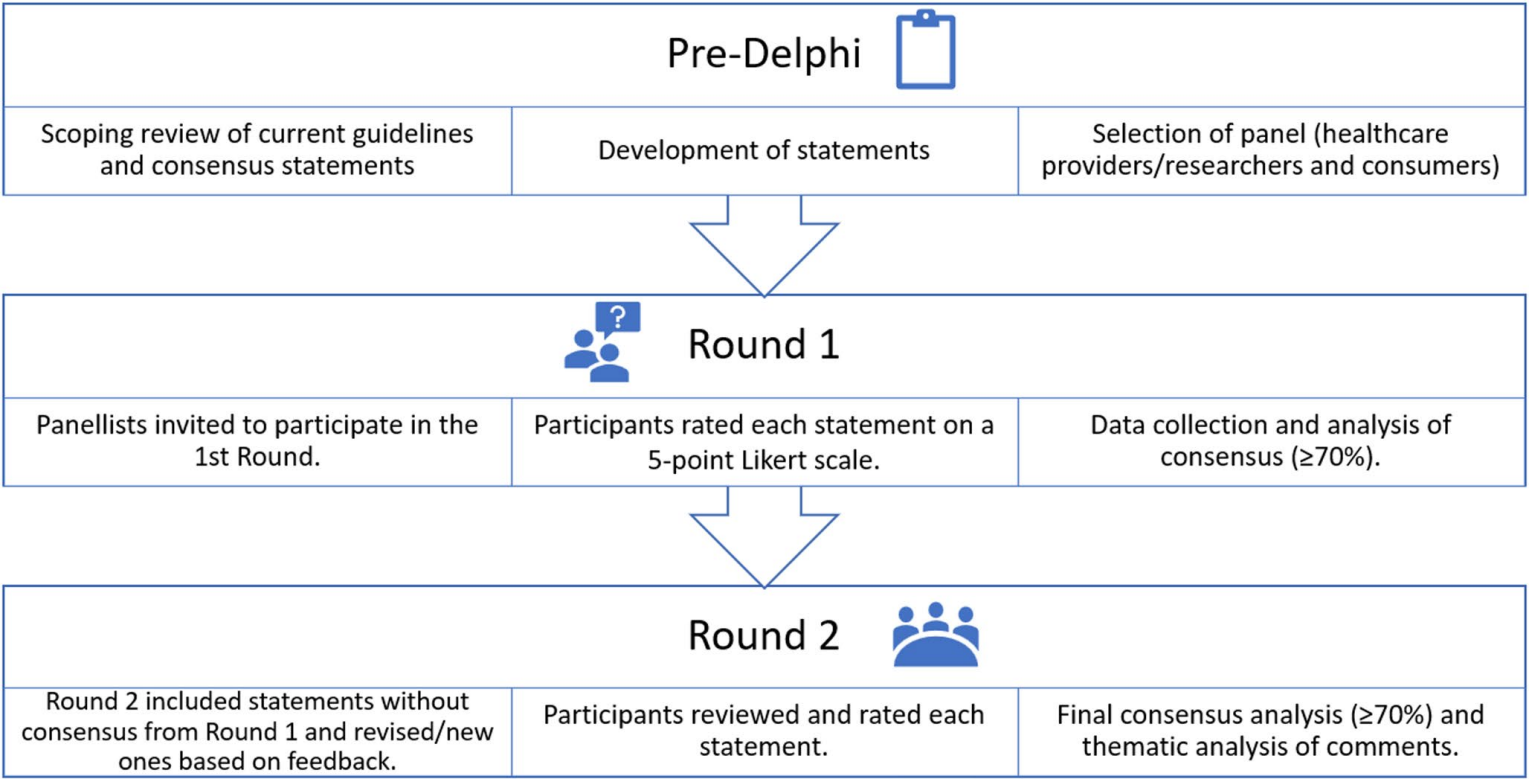
Modified Delphi technique

A modified Delphi technique was used to elicit consensus regarding genetic testing and genetic counselling in PCa (Fig. 1). A modified technique typically has more focussed input and fewer rounds, more concerned with refining and clarifying specific ideas than a traditional Delphi which tends to have more open-ended questions and be concerned with generating a broad range of ideas and perspectives [11]. We chose the secure web platform, REDCap, hosted by the University of Queensland, to manage the Delphi Survey.

### Delphi statements

The initial step of the Delphi study process was to develop the statements for inclusion in the Delphi survey. Strategies synthesized from international guidelines and consensus statements generated by our scoping review [9] informed the development of statements. In discussion with our multidisciplinary Steering Committee, seven domains of interest were identified and the statements classified under these: i). who should be tested; ii). which genes should be tested; iii) how genetic testing results inform prostate specific antigen testing; iv) how genetic testing results inform the management of prostate cancer; v). when genetic counselling should be provided; vi). who should provide genetic counselling; and vii). how genetic counselling should be carried out. The initial survey was piloted with the Steering Committee, during which an iterative feedback process was carried out to enhance the structure and readability of the statements as well as to test the accessibility of the platform.

A comprehensive survey incorporating the full seven domains, comprising 55 statements was administered to healthcare providers and researchers, while a shorter version incorporating only four domains (1, 5, 6, 7) comprising 34 statements was provided to consumers (patient, family member, carers or non-health provider/researcher). This approach was taken to account for the specialized clinical knowledge required to respond to certain statements (domains 2, 3, 4), particularly those pertaining to which genes should be tested and how the genetic testing results inform PSA (prostate specific antigen) testing and the management of PCa.

### Recruitment of Delphi participants

We used the professional networks of the Steering Committee and relevant professional associations (e.g., PCFA, ANZUP, ASGC) to recruit respondents for the Delphi survey. A purposive sampling approach was employed which involved emailing invitations to participate to suitably qualified stakeholders (medical oncologists, radiation oncologists, urologists, geneticists, genetic counsellors, prostate cancer researchers, prostate cancer specialised nurses, patients and carers). The email included a brief overview of the study, a participant information sheet and a link to the online survey. To ensure consistency, members of the Steering Committee invited their colleagues to participate by forwarding the same email to their professional networks. When clinicians were slow to respond to the invitations to participate in the research, a QR code was created with a link to the Delphi survey and the participant information statement in REDCap. This was then displayed on a PowerPoint slide before Steering Committee members presented at the Australia and New Zealand Urogenital and Prostate Cancer Trials Group (ANZUP) annual scientific meeting in Melbourne in 2023. The Prostate Cancer Foundation of Australia (PCFA) disseminated this email through their mailing lists which included clinicians, allied health practitioners, nurses, researchers, patients and carers. The same information was also disseminated through USANZ UroNews and the Australasian Society of Genetic Counsellors (ASGC) newsletter.

### Delphi rounds

Two surveys were administered via REDCap between February and April 2023 [12, 13]. Each round was open for a minimum of two weeks. In line with the conditions of HREC approval, completion of the survey implied consent, which was explained to participants in the survey instructions when they commenced the survey.

In Round 1, participant demographics data such as gender, age, profession or role, speciality (for healthcare providers), was collected. Participants were then asked to rate the statements, using a 5-point Likert scale ('strongly agree', 'agree', 'neutral', 'disagree', 'strongly disagree'), based on its benefit and feasibility. Additionally, a free-text response was provided to the participant within each of the survey domains, offering them the opportunity to provide feedback on statements and/or written justification for their choices.

Statements that reached consensus (≥ 70%) were excluded from Round 2, while statements that did not reach consensus in Round 1 were either carried forward or revised based on the feedback received. Five new statements were also included in Round 2 based on the comments received in Round 1. For Round 2, individual survey links were sent to respondents using the email address provided in Round 1. Participants were asked to rate the statements using the same method as described in Round 1.

### Statistical analysis

Data from REDCap were exported to Microsoft Excel for statistical analysis. Descriptive statistics were utilised to describe participants’ demographic characteristics and their ratings for each statement. While consensus in Delphi studies is widely debated [14], for the purposes of this Delphi panel with a diverse range of participants, positive consensus was defined a priori as ≥ 70% of participants indicating 'strongly agree' or 'agree' with a statement, while negative consensus for a statement was defined as ≥ 70% of participants indicating 'strongly disagree' or 'disagree'. Furthermore, a median score and interquartile range (IQR) were calculated for each statement to provide insight into the distribution or spread of scores [15]. The interquartile range (IQR) provides a measurement of how dispersed or spread out the middle 50 per cent of the data set is and is therefore not sensitive to outliers, so can be used to show strength of consensus. An IQR of zero signifies a perfect consensus among panel members, whereas a higher IQR indicates a greater dispersion of the data.

Thematic analysis, a systematic approach to qualitative data coding, was also conducted to interpret participants’ free-text responses and gain some understanding of the reasons behind their agreement or disagreement with the statements [16].

### Ethics

Ethical approval for this study was granted by the Health and Behavioural Science Research Committee at the University of Queensland (2022/HE001998).

## Results

### Participant demographics

Thirty-six HP/Rs participated in Round 1 of the Delphi survey, representing seven different occupations; 72 per cent of whom identified a public hospital as their primary area of practice. The second participant cohort was consumers (n = 27), 81 per cent of whom were patients. In Round 2, 25 HP/Rs and 21 consumers completed the Delphi survey. When HP/Rs rated their knowledge of genetic testing out of 5, the mean score was higher than that of consumers (Table 1).

**Table 1:** Participant Demographics

Characteristics	n (%)
*Healthcare professional/researchers (N = 36)*	
Gender (male)	18 (50%)
*Occupation *	
Urologist	7 (19%)
Oncologist	10 (28%)
Geneticist	2 (6%)
Clinical Nurse Specialist (CNS)	6 (17%)
Genetic counsellor	7 (19%)
Researcher	2 (6%)
Other	2 (6%)
*Primary area of practice*	
Public hospital	26 (72%)
Private hospital	13 (36%)
Public clinic	3 (8%)
Private clinic	8 (22%)
Specialist prostate cancer clinic	2 (6%)
At patient's home	1 (3%)
Other	4 (11%)
Self-rated knowledge	3.5
*Consumers (N = 27)*	
Gender (male)	24 (89%)
*Role*	
Patient	22 (81%)
Family member	1 (4%)
Carer	2 (7%)
Other	2 (7%)
Self-rated knowledge	2.3

### Genetic testing

#### Healthcare providers/researchers

In Round 1, 21 out of the total 55 statements (38%) in the Delphi survey achieved consensus amongst HP/Rs. Of the 36 statements related to genetic testing, consensus was achieved for six statements (see Table 2 and Supplementary Information Table 1 for details).

**Table 2:** Level of consensus for genetic testing among healthcare providers/researchers

Statements	Agreement (%)	Median (IQR)	Consensus
*i. Which men should be considered for genetic testing?*			
Men without PCa and a family history of high-risk hereditary (germline) cancer predisposition genes	**100**	1 (0)	Consensus*
Men without PCa and a family history of multiple cancers on the same side of the family	**75**	2 (1)	Consensus*
Men without PCa and one or more close blood relatives with a PCa diagnosis at < 60 years	**75**	2 (1)	Consensus*
Men without PCa and one or more close blood relatives with metastatic (advanced) PCa	40	3 (2)	–
Men without PCa and one or more close blood relatives who died of PCa	52	2 (2)	–
Men with PCa and a family history of HBOC syndrome (including breast, ovarian, pancreatic, prostate)	**97**	1 (0)	Consensus*
Men with PCa and a family history of PCa	**76**	2 (1)	**Consensus**
Men with PCa and a family history of Lynch Syndrome (colorectal, upper gastrointestinal tract, endometrial, ovarian, pancreatic prostate, upper urinary tract cancers and sebaceous adenocarcinomas)	**75**	2 (1)	Consensus*
Men with non-metastatic PCa (cancer that has not spread outside the prostate gland) and Ashkenazi Jewish ancestry	**76**	2 (1)	**Consensus**
Men with non-metastatic PCa and Grade Group 4 (Gleason score 8 when cells look abnormal and may grow at a moderate to fast rate) or above	28	3 (2)	–
Men with non-metastatic PCa and advanced disease (T3—cancer that has spread outside the prostate gland, but not progressed to other parts of the body)	20	4 (1)	–
Men with non-metastatic PCa and intraductal/ductal pathology (aggressive cancer found in the prostatic ducts)	24	3 (1)	–
All men with metastatic PCa should have somatic genetic testing (testing for acquired [not inherited] mutations in the tumour tissue)	64	2 (2)	–
All men with metastatic PCa should have germline genetic testing (testing for inherited genetic mutations via cheek swab, spit or blood sample)	56	2 (1)	–
Men with metastatic PCa and somatic genetic testing results showing mutation in cancer risk genes should have germline genetic testing (factoring in personal and family history)	**89**	2 (1)	Consensus*
*v. Which genes should be tested for based on clinical/familial scenarios?*			
Men with family history of PCa should be tested for HOXB13	13	3 (1)	–
Men with family history of HBOC syndrome (including pancreatic, prostate and melanoma) should be tested for *BRCA1 and BRCA2*	57	2 (1)	–
Men with family history of HBOC syndrome (including pancreatic, prostate and melanoma) should be tested for *BRCA1 and BRCA2* (particularly if there is a history of early diagnosis, multiple such cancers on the same side of the family and/or Ashkenazi Jewish ancestry)	61	2 (2)	–
Men with family history of hereditary Lynch syndrome (including colorectal, stomach, endometrial, liver, kidney, etc.) should be tested for *DNA MMR genes (MLH1, MSH2, MSH6, PMS2 and EPCAM)*	43	3 (1)	–
Men with family history of multiple cancers (e.g. HBOC syndrome; Lynch syndrome; colorectal cancer, etc.) on the same side of the family should be considered for comprehensive large/broad panel testing	57	2 (1)	–
Men with PCa and two or more close blood relatives on same side of the family with HBOC syndrome (including pancreatic, prostate and melanoma) should be tested for *BRCA1 and BRCA2*	**83**	2 (1)	**Consensus**
Men with PCa and two or more close blood relatives on same side of the family with Lynch syndrome should be tested for *DNA MMR genes (MLH1, MSH2, MSH6, PMS2 and EPCAM)*	52	2 (1)	–
Men with metastatic PCa should have somatic next generation sequencing	**70**	2 (1)	**Consensus**
Men with metastatic PCa should have germline testing for *BRCA1, BRCA2, DNA MMR genes (MLH1, MSH2, MSH6, PMS2 and EPCAM) and ATM*	57	2 (2)	–
Men with metastatic PCa should have confirmatory germline testing for *BRCA2* only after a pathogenic variant or likely pathogenic variant is detected by somatic testing	35	3 (2)	–
Men with metastatic PCa should have confirmatory germline testing for *BRCA2, BRCA1, DNA MMR genes (MLH1, MSH2, MSH6, PMS2 and EPCAM) and ATM* after a pathogenic variant is detected by somatic testing	**73**	2 (2)	**Consensus**
Men with metastatic PCa should be offered germline testing based on the allele frequency of the somatic testing (frequency of 40–60% or germline testing may not identify any pathogenic variants)	36	3 (2)	–
Men with metastatic castration resistant PCa should have somatic testing for *BRCA1 and BRCA2*	**82**	2 (1)	**Consensus**
Men with metastatic castration-resistant PCa should have somatic and germline testing for *BRCA1, BRCA2, PALB2 and ATM*	68	2 (1)	–
Men with metastatic castration-resistant PCa should have genetic testing regardless of family history (a multigene panel including at least *BRCA1, BRCA2 and MMR genes [MLH1, MSH2, MSH6, PMS2 and EPCAM]*) to inform treatment	**73**	2 (1)	**Consensus**
*vi. How would genetic testing results inform PSA testing for prostate cancer?*			
Men without PCa with a pathogenic variant in a gene known to cause PCa should have PSA testing discussions	**100**	1 (1)	**Consensus**
Men without PCa with no detected pathogenic variants and a family history of PCa, particularly if early age at diagnosis, should have PSA testing discussions	**95**	2 (1)	**Consensus**
Men with localised PCa should have PSA testing discussions if they have a pathogenic variant in *BRCA2, BRCA1, HOXB13, ATM, DNA MMR*, (particularly if they have a history of multiple cancers in the same side of the family, early age at diagnosis or Ashkenazi Jewish ancestry)	64	2 (2)	–
*vii. How should genetic testing results inform management of PCa?*			
Of all genes on multigene panels for non-metastatic PCa, only *BRCA2* should be factored into management discussions (e.g., active surveillance)	6	4 (1)	–
Of all genes on multigene panels for metastatic PCa, only *BRCA2 and BRCA1* should be factored into management discussions	12	4 (1)	–
Of all genes on multigene panels for metastatic castration-resistant PCa, only *BRCA2, BRCA1 and ATM* should be factored into management discussions	21	3 (1)	–

Bold indicate consensus ≥ 70%

^*^ indicates consensus achieved in 1st Round of Delphi Panel

HBOC = hereditary breast and ovarian cancer; PCa = prostate cancer; PSA = Prostate Specific Antigen

In Round 2, after questions were revised and new ones added based on comments provided by HP/Rs in Round 1, nine of the 28 statements (32%) in the survey relating to genetic testing achieved consensus. Fifteen statements in Round 2 related to which genes should be tested based on clinical/familial scenarios and even after revisions and the addition of a statement based on respondent comments, only five (33%) statements achieved consensus.

#### Consumers

In Round 1, all statements (100%) relating to who should receive genetic testing (n = 15) achieved consensus amongst consumers. Therefore, there was no Round 2 for consumers with regard to genetic testing.

### Genetic counselling

#### Healthcare providers/researchers

In Round 1, 13 of 19 statements (68%) relating to genetic counselling achieved consensus. Seven of the 13 statements that achieved very high levels of consensus (≥ 90%) related to when and how genetic counselling should be conducted (Supplementary Information Table 1).

In Round 2, two of the eight statements relating to genetic counselling achieved consensus. Eighty-three per cent consensus was achieved for a new statement derived from Round 1 comments relating to a partial mainstream consent pathway for the provision of genetic counselling. Partial mainstream consent is where medical oncologists or other HPs perform consent for germline testing then fast track positive patients to genetic counselling [17]. The second was a statement about provision of genetic counselling by psychologists, counsellors and socials workers that also achieved 83 per cent negative consensus (Supplementary Information Table 1).

#### Consumers

In Round 1, 16 of 19 statements (84%) relating to genetic counselling achieved consensus. Of these, six statements achieved very high consensus (≥ 90%) and these related to when genetic counselling should be provided (n = 3), and how it should be carried out (n = 3) (Supplementary Information Table 2).

Round 2 comprised four statements, a new statement and a revised statement referring to who should conduct genetic counselling based on comments from Round 1 and the remaining two statements that did not achieve consensus. Both the new statement (90%) and the revised statement (76%) achieved consensus. The level of consensus for the remaining two statements, one regarding a psychologist, counsellor or social worker providing genetic counselling and the other the effectiveness of genetic counselling conducted via video conferencing/telehealth, remained much the same across rounds (41–43% and 52–52%, respectively) (Supplementary Information Table 2).

### Strength of consensus

Of the 15 of 36 statements for HP/Rs that achieved consensus on genetic testing, 14 showed strong consensus (IQR  0-1) with only 1 statement scoring an IQR of 2 (Table 2). For consumers, of the 15 of 15 statements that achieved consensus on genetic testing, 14 showed strong consensus (IQR 0–1) with only one statement scoring an IQR of 2.

Of the 27 statements for HP/Rs that achieved consensus on genetic counselling, all showed strong consensus (IQR 0–1). Of the 18 statements for consumers that achieved consensus on genetic counselling, 16 statements showed strong consensus (IQR 0–1) with two statements scoring an IQR of 1.5.

### Comparison of findings for healthcare providers and consumers

There were a number of statements common to both cohorts of respondents (HP/Rs and consumers) where responses conflicted (Supplementary Information Tables 1 and 2). When it came to which men should be considered for genetic testing in Round 1, only six statements achieved consensus in the HP/R cohort, whereas all statements achieved consensus in the consumer cohort. In relation to genetic counselling, a number of statements generated conflicting results (Table 3). For example, there were conflicting results in relation to whether genetic counselling should be provided for those with a negative genetic testing result with agreement from 74 per cent of consumers and only 50 per cent of HP/Rs. There were more conflicting responses to the topic of who should provide genetic counselling with 50 per cent of statements recording very different responses across the two cohorts. While consumers agreed to seven out of eight suggested providers of genetic counselling, HP/Rs agreed to only three; genetic counsellors, qualified allied health professionals or nurses and the partial mainstream consent pathway. Interestingly, both cohorts did not agree to genetic counselling being conducted by psychologists, counsellors or social workers. In relation to how genetic counselling is carried out, there was conflict between the cohorts in two statements—these were in relation to face to face versus videoconferencing or telehealth. While 85 per cent of consumers agreed that genetic counselling should be conducted face to face, only 17 per cent of HP/Rs agreed with this statement. Similarly, 86 per cent of HP/Rs agreed genetic counselling can be conducted effectively using videoconferencing or telehealth whereas only 52 per cent of consumers agreed with this statement.

**Table 3:**
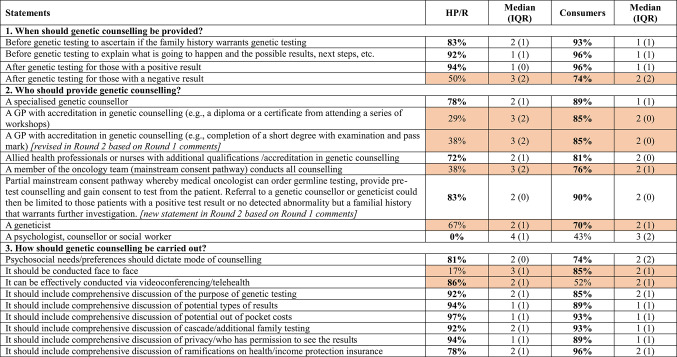
Level of consensus (agreement) for genetic counselling among both healthcare provider/researchers and consumers

**Bold** indicates consensus achieved (≥ 70% agreement or negative consensus where there was ≥ 70% disagreement)

**Shaded** items indicate conflicting responses between healthcare providers and consumers

HP/R = healthcare provider/researcher, IQR = interquartile ranges

### Qualitative evaluation of comments

Coding and thematic analysis of qualitative data from the comments section of the Delphi survey revealed a number of recurrent themes in the responses from the HP/R cohort. *Specificity* arose numerous times in response to the statements and was used by genetic counsellors, geneticists, researchers and oncologists. Specificity referred to the need for more specific information such as costs and benefits, allele frequency, capacity to test, lack of resources, etc., or more nuanced statements than those provided in the survey before they could make a decision. Some respondents chose a neutral response as a result.

*Specialized knowledge* was a theme that occurred in 80 per cent of comments relating to Domain 3 in the Delphi survey statements about who should provide genetic counselling. In 60 per cent of comments, pre-test counselling was viewed as less dependent on genetic expertise but still requiring a clinician or sufficiently trained oncology nurse or allied healthcare provider, followed by post-test genetic counselling conducted by a specialized genetic counsellor.

With regard to how genetic counselling should be carried out, *men without a diagnosis* arose in 24 per cent of comments, in relation to the importance of predictive testing and the impact on income protection and life insurance. *Life insurance* was mentioned in 25 per cent of all comments across questions. *Face to face/telehealth* was mentioned as equally appropriate for genetic counselling purposes in 35 per cent of comments; *individual patient need or convenience* arose in 20 per cent of comments in relation to telehealth. One oncologist commented that telehealth is inappropriate in regional, rural and remote areas due to lack of infrastructure or access and for the elderly due to ignorance of technology in 80 per cent of cases.

*Any man/all men* occurred frequently in comments (50%) relating to how genetic testing results would inform PSA testing, particularly in relation to high risk and family history. Forty per cent of comments mentioned *all men* regardless of status should have access to PSA screening and discussions. *Specific genes* were mentioned in 25 per cent of comments.

With regard to the question of how genetic testing should inform disease management, 30 per cent of respondents said to inform *targeted treatment*; 20 per cent said to inform PSA screening and 20 per cent commented they had insufficient knowledge to respond to the statement and chose a neutral response.

Only 11 consumer respondents made comments across the two rounds of the Delphi study. Consumer comments were often accounts of personal experiences or family history. One respondent expressed concern regarding the impact of genetic testing on insurance for unaffected siblings. Two expressed their belief that genetic counselling should be held face to face, if feasible. Two comments referred to the need for specialized knowledge when conducting genetic counselling and one of these noted the lack of genetic counsellors.

## Discussion

This study identified key strategies for genetic testing and genetic counselling in PCa using a modified Delphi-technique. With regard to who should receive genetic testing, there were eight target population groups identified, which all achieved ≥ 75% consensus. The target groups for men without PCa included those with a family history of i) high-risk hereditary (germline) genes; ii) multiple cancers on the same side of the family; and iii) one or more blood relatives with an early PCa diagnosis (at < 60 years). For men with PCa, the target groups were those men with a family history of iv) PCa; v) HBOC syndrome; or vi) Lynch syndrome. The final two population groups were men with vii) Ashkenazi Jewish ancestry or viii) metastatic PCa and somatic genetic testing results showing mutation in cancer risk genes. These findings align with major international guidelines by organizations such as the National Comprehensive Cancer Network (NCCN), the European Society for Medical Oncology (ESMO)[9] and the American Society of Clinical Oncology (ASCO)[18]. Both germline and somatic testing has recently been recommended for mPCa [18, 19] and even universal germline genetic testing for all patients diagnosed with PCa [20].

There was a high level of consensus for genetic counselling stating that 1) genetic counselling be provided prior to genetic testing to ascertain family history and to explain the implications of a positive test result; 2) genetic counselling be performed post-testing in those with a positive test result. Additionally, there were high levels of agreement that both specialized genetic counsellors and medical oncologists could provide genetic counselling, the latter via a partial mainstream consent pathway. HP/Rs also agreed that counselling could be carried via video conferencing/telehealth and should include a comprehensive discussion on the purpose of testing, cascade testing, types of results, potential out-of-pocket costs and privacy of test results. Genetic counselling is broadly accepted as a necessary part of the process of genetic testing, however, the detail in international guidelines ranges from recommendations for pre-testing and post-testing genetic counselling, eligibility and process, along with a list of topics to be covered [21–23]; a general recommendation that genetic counselling is an essential and mandatory part of genetic testing but provide little other detail [24–35], to no mention of genetic counselling at all [36–39]. Only the Swedish guidelines cite concern for psychological impact on the patient and their family [36].

The social implications of genetic testing are far reaching and include potential impacts on family relationships, concerns about privacy around genetic information and the risk of discrimination in employment or insurance, social stigma, the need for informed decision-making and support systems when interpreting test results which could potentially affect a person’s sense of identity, psychological wellbeing, employment, security and future plans. Ramifications include a need for informed consent, genetic counselling and legal protections [40, 41].

The overall consensus on statements for genetic testing and genetic counselling was higher among consumers compared to HP/Rs. One hundred per cent of consumers achieved consensus on statements on genetic testing and 84 per cent agreed on statements on genetic counselling from Round 1 of the survey. Further, two of the four statements on genetic counselling that were revised or additionally developed for Round 2 of the Delphi surveys achieved high levels of consensus (≥ 75%). Consensus for consumers was markedly different to that of HP/R, suggesting consumers wanted universal access to genetic testing, with higher levels of consensus for testing in men with family history and men with metastatic disease. With genetic counselling, consumers were seemingly more concerned with getting counselling and not so much about who provides it when compared to HP/Rs.

Our results of lower consensus for genetic testing and genetic counselling in PCa among HP/Rs compared to consumers could be explained by several factors. First, the disinclination for genetic testing among HP/Rs could be influenced by trends in screening practices for PCa based on PSA. PCa is often considered to be an over diagnosed and overtreated condition and after the US Preventive Services Task Force (USPSTF) guidelines recommendation against routine PSA screening of all men in 2008, there has been a steady decline in PCa incidence, suggesting lower utilization of PCa screening using PSA among health care providers [42, 43]. Second, there is still considerable uncertainty in terms of the genetic variants to be included in panel testing and their overall pathogenicity. The presence of a pathogenic germline variant does not always lead to increased PCa expression or disease progression. Factors such as penetrance and the distinct mechanisms involved in DNA instability as a result of the pathogenic genetic variation play a vital role in disease expression. It is plausible that compared to consumers, HP/Rs are more cognizant of the issues surrounding genetic testing (both in terms of the science behind genetic variation and more practical concerns like patient uptake, lack of reimbursement, lack of counsellors, lack of testing resources and the need for clinician education) and these factors could have influenced their rates of consensus for genetic testing and genetic counselling [41, 44].

### Strengths and limitations

Our results have several strengths. The development of this Delphi Panel study was based on a scoping review of international guidelines which involved a synthesis of existing statements from those guidelines. We had a comprehensive expert steering committee comprising oncologists, urologists, geneticists, cancer psychologists, PCa researchers and health economists who supported the development of the Delphi Panel from the formation of the survey statements and their revision to participation in a pilot survey before the survey went live. Through steering committee networks and professional affiliations, we were able to involve healthcare provider and research stakeholders across the spectrum of PCa care and genetic testing as well as a strong contingent of committed consumers, which meant the perspective of those with the disease or those caring for men with the disease could be heard. Including a comment section in each of the domains in the survey improved the quality and accuracy of the statements (via revision for Round 2) as well as allowing qualitative analysis which enabled the sentiment behind the responses to be better understood. Consensus in Delphi studies is widely debated, yet 75 per cent consensus was considered the median threshold in a previous systematic review [14]. While our consensus threshold was 70 per cent, we achieved 75 per cent consensus in 33 per cent of statements on genetic testing and 62 per cent of statements on genetic counselling from HPs/Rs. From consumers, we achieved 75 per cent consensus in 93 per cent of statements on genetic testing and 65 per cent of statements on genetic counselling. In addition, our work complements the work of eviQ. The exploration of pre- and post-test counselling models and the integration of consumer perspectives are strengths that enhance the manuscript’s relevance and potential impact.

The study also had its limitations. It is possible that our HP/R cohort with only 72 per cent having specialist clinical or genetics knowledge may have contributed to more neutral or disagree responses due to a lack of knowledge, and therefore, a lower proportion of statements that achieved consensus. The small representation of clinical geneticists and genetic counsellors, while consistent with the current Australian workforce and the growing mainstreaming of testing through oncology services, may have limited perspectives on counselling and familial risk assessment.

A further limitation is that some Delphi panel responses diverged from current clinical practice or contained ambiguity in interpretation. For example, Medicare-funded testing (MBS items 73357 and 73297) applies only when a pathogenic or likely pathogenic variant has been identified in a biological relative, whereas some panel statements referred to testing based solely on family history. Similarly, the lack of consensus regarding testing in men with non-metastatic prostate cancer and intraductal or ductal pathology, and ambiguity in statements referencing Lynch or HBOC syndromes, contrasts with current evidence and eviQ recommendations. These discrepancies likely reflect differences in interpretation or awareness among participants rather than disagreement with established best practice. There is also the possibility that statements worded differently may have achieved greater consensus and that a full Delphi study which involved a third discussion round may also have yielded different results.

There is currently a lack of economic evaluation and cost-effectiveness evidence for genetic testing in PCa [45]. This evidence is essential in deciding who should be tested, how they should be tested and the most appropriate management pathway [46, 47]. A standardized approach is vital in determining the value of genetic testing for PCa. However, there is also a need for flexibility and innovation in the delivery of genetic testing and counselling based on the specific capacity of different regions to deliver genetic testing as per the major international guidelines.

## Conclusion

This Delphi Panel study was the first in Australia to interrogate genetic testing recommendations from current international genetic testing for PCa guidelines. Our findings affirm the inclination of both HP/Rs and consumers for testing those with family history of HBOC syndrome, inclusion of *BRCA1/2* and *MMR* genetic variants in testing and genetic counselling prior to testing, either from specialized genetic counsellors or medical oncologists. Our study also highlighted a lack of resources (testing and access to genetic counsellors) and the need for greater clinician education. There is also a need to evaluate the effectiveness and value for money of genetic testing strategies before implementation in practice.

## Supplementary Information

Below is the link to the electronic supplementary material.

Supplementary Material 1

## Data Availability

All data generated or analysed during this study are included in this published article and its supplementary information files.
