# Supplementary material for: Delphi panel consensus on genetic testing for prostate cancer in Australia: Whom to test and how?
Source: Fam Cancer. 2026 Jan 24;25(1):12. doi: 10.1007/s10689-025-00528-x (PMC12831670; doi:10.1007/s10689-025-00528-x)
Supplement: Supplementary file 1 — Supplementary Material 1 [file 10689_2025_528_MOESM1_ESM.docx]

**Supplementary Table 1: Level of consensus for genetic testing and genetic counselling among healthcare provider/researchers (Round 1 and Round 2)**

| **Round** | **Statements** | **Agree** | **Neutral** | **Disagree** | **Consensus** |
| --- | --- | --- | --- | --- | --- |
| **1. Which men should be considered for genetic testing?** | | | | | |
| 1 | Men without PCa and a family history of high-risk hereditary (germline) cancer predisposition genes | **100%** | 0% | 0% | **Consensus*** |
| 1 | Men without PCa and a family history of multiple cancers on the same side of the family | **75%** | 11% | 14% | **Consensus*** |
| 1 | Men without PCa and one or more close blood relatives with a PCa diagnosis at < 60 years | **75%** | 8% | 17% | **Consensus*** |
| 1 | Men with one or more close blood relatives with metastatic (advanced) PCa | 50% | 31% | 19% | - |
| 2 | Men without PCa and one or more close blood relatives with metastatic (advanced) PCa *[revised based on feedback]* | 40% | 32% | 28% | - |
| 1 | Men with one or more close blood relatives who died of PCa | 50% | 31% | 19% | - |
| 2 | Men without PCa and one or more close blood relatives who died of PCa *[revised based on feedback]* | 52% | 20% | 28% | - |
| 1 | Men with PCa and a family history of Hereditary Breast and Ovarian Cancer (HBOC) syndrome (including breast, ovarian, pancreatic, prostate) | **97%** | 3% | 0% | **Consensus*** |
| 1 | Men with PCa and a family history of PCa | 61% | 36% | 3% | - |
| 2 | Men with PCa and a family history of PCa *[carried forward from Round 1]* | **76%** | 12% | 12% | **Consensus** |
| 1 | Men with PCa and a family history of Lynch Syndrome (colorectal, upper gastrointestinal tract, endometrial, ovarian, pancreatic prostate, upper urinary tract cancers and sebaceous adenocarcinomas) | **75%** | 19% | 6% | **Consensus*** |
| 1 | Men with non-metastatic PCa (cancer that has not spread outside the prostate gland) and Ashkenazi Jewish ancestry | 58% | 31% | 11% | - |
| 2 | Men with non-metastatic PCa (cancer that has not spread outside the prostate gland) and Ashkenazi Jewish ancestry *[carried forward from Round 1]* | **76%** | 16% | 8% | **Consensus** |
| 1 | Men with non-metastatic PCa and Grade Group 4 (Gleason score 8 when cells look abnormal and may grow at a moderate to fast rate) or above | 28% | 33% | 39% | - |
| 2 | Men with non-metastatic PCa and Grade Group 4 (Gleason score 8 when cells look abnormal and may grow at a moderate to fast rate) or above *[carried forward from Round 1]* | 28% | 24% | 48% | - |
| 1 | Men with non-metastatic PCa and advanced disease (T3 - cancer that has spread outside the prostate gland, but not progressed to other parts of the body) | 22% | 39% | 39% | - |
| 2 | Men with non-metastatic PCa and advanced disease (T3 - cancer that has spread outside the prostate gland, but not progressed to other parts of the body) *[carried forward from Round 1]* | 20% | 20% | 60% | - |
| 1 | Men with non-metastatic PCa and intraductal/ductal pathology (aggressive cancer found in the prostatic ducts) | 28% | 42% | 31% | - |
| 2 | Men with non-metastatic PCa and intraductal/ductal pathology (aggressive cancer found in the prostatic ducts) *[carried forward from Round 1]* | 24% | 32% | 44% | - |
| 1 | All men with metastatic PCa should have somatic genetic testing (testing for acquired [not inherited] mutations in the tumour tissue) | 64% | 22% | 14% | **-** |
| 2 | All men with metastatic PCa should have somatic genetic testing (testing for acquired [not inherited] mutations in the tumour tissue) *[carried forward from Round 1]* | 64% | 22% | 14% | - |
| 1 | All men with metastatic PCa should have germline genetic testing (testing for inherited genetic mutations via cheek swab, spit or blood sample) | 33% | 33% | 33% | - |
| 2 | All men with metastatic PCa should have germline genetic testing (testing for inherited genetic mutations via cheek swab, spit or blood sample) *[carried forward from Round 1]* | 56% | 20% | 24% | - |
| 1 | Men with metastatic PCa and somatic genetic testing results showing mutation in cancer risk genes should have germline genetic testing (factoring in personal and family history) | 89% | 8% | 3% | **Consensus*** |
| **2. Which genes should be tested for based on clinical/familial scenarios?** | | | | | |
| 1 | Men with family history of PCa should only be tested for *HOXB13* | 9% | 41% | 50% | - |
| 2 | Men with family history of PCa should be tested for *HOXB13* *[revised based on feedback]* | 13% | 43% | 43% | - |
| 1 | Men with family history of HBOC syndrome should only be tested for *BRCA1 and BRCA2* | 21% | 32% | 47% | - |
| 2 | Men with family history of hereditary breast and ovarian cancer (HBOC) syndrome (including pancreatic, prostate and melanoma) should be tested for *BRCA1 and BRCA2* *[revised based on feedback]* | 57% | 26% | 17% | - |
| 2 | Men with family history of hereditary breast and ovarian cancer (HBOC) syndrome (including pancreatic, prostate and melanoma) should be tested for *BRCA1 and BRCA2* (particularly if there is a history of early diagnosis, multiple such cancers on the same side of the family and/or Ashkenazi Jewish ancestry) *[revised based on feedback]* | 61% | 22% | 17% | - |
| 1 | Men with family history of Lynch Syndrome should only be tested for *DNA MMR genes (MLH1, MSH2, MSH6, and PMS2)* | 34% | 37% | 29% | - |
| 2 | Men with family history of hereditary Lynch syndrome (including colorectal, stomach, endometrial, liver, kidney, etc.) should be tested for *DNA MMR genes (MLH1, MSH2, MSH6, PMS2 and EPCAM)* *[revised based on feedback]* | 43% | 35% | 22% | - |
| 1 | Men with family history of multiple cancers on the same side of the family should be considered for comprehensive large/broad panel testing. | 66% | 20% | 14% | - |
| 2 | Men with family history of multiple cancers (e.g. HBOC syndrome; Lynch syndrome; colorectal cancer, etc.) on the same side of the family should be considered for comprehensive large/broad panel testing *[revised based on feedback]* | 57% | 22% | 22% | - |
| 1 | Men with PCa and two or more close blood relatives on same side of the family with HBOC should only be tested for *BRCA2* | 12% | 29% | 59% | - |
| 1 | Men with PCa and two or more close blood relatives on same side of the family with HBOC should only be tested for *BRCA1 and BRCA2* | 21% | 24% | 56% | - |
| 2 | Men with PCa and two or more close blood relatives on same side of the family with hereditary breast and ovarian cancer (HBOC) syndrome (including pancreatic, prostate and melanoma) should be tested for *BRCA1 and BRCA2* *[revised based on feedback]* | **83%** | 9% | 9% | **Consensus** |
| 1 | Men with PCa and two or more close blood relatives on same side of the family with Lynch Syndrome should only be tested for *DNA MMR genes* | 21% | 35% | 44% | - |
| 2 | Men with PCa and two or more close blood relatives on same side of the family with Lynch syndrome should be tested for *DNA MMR genes (MLH1, MSH2, MSH6, PMS2 and EPCAM)* *[revised based on feedback]* | 52% | 30% | 17% | - |
| 1 | Men with no-metastatic PCa should only be tested for *BRCA2 and ATM* | 3% | 35% | 62% | - |
| 1 | Men with metastatic PCa should only have somatic next generation sequencing | 9% | 44% | 47% | - |
| 2 | Men with metastatic PCa should have somatic next generation sequencing *[revised based on feedback]* | **70%** | 22% | 9% | **Consensus** |
| 1 | Men with metastatic PCa should have germline testing for *BRCA1, BRCA2, DNA MMR genes and ATM* | 32% | 53% | 15% | - |
| 2 | Men with metastatic PCa should have germline testing for *BRCA1, BRCA2, DNA MMR genes (MLH1, MSH2, MSH6, PMS2 and EPCAM) and ATM [revised based on feedback]* | 57% | 17% | 26% | - |
| 1 | Men with metastatic PCa should have confirmatory germline testing for *BRCA2* only after a pathogenic mutation is detected by somatic testing. | 38% | 41% | 21% | - |
| 2 | Men with metastatic PCa should have confirmatory germline testing for *BRCA2* only after a pathogenic variant or likely pathogenic variant is detected by somatic testing *[revised based on feedback]* | 35% | 26% | 39% | - |
| 1 | Men with metastatic PCa should have confirmatory germline testing for *BRCA2, BRCA1, DNA MMR* genes (*MLH1, MSH2, MSH6, PMS2 and EPCAM*) and *ATM* after a pathogenic variant is detected by somatic testing | **71%** | 24% | 6% | **Consensus*** |
| 2 | Men with metastatic PCa should be offered germline testing based on the allele frequency of the somatic testing (frequency of 40-60% or germline testing may not identify any pathogenic variants) *[new statement]* | 36% | 36% | 27% | - |
| 1 | Men with metastatic castration resistant PCa should have somatic testing for *BRCA1 and BRCA2* only | 26% | 35% | 38% | - |
| 2 | Men with metastatic castration resistant PCa should have somatic testing for *BRCA1 and BRCA2* *[revised based on feedback]* | **82%** | 9% | 9% | **Consensus** |
| 1 | Men with metastatic castration resistant PCa should have somatic testing for *ATM* only | 3% | 29% | 68% | - |
| 1 | Men with metastatic castration-resistant PCa should have somatic and germline testing for *BRCA1, BRCA2, PALB2 and ATM* | 44% | 41% | 15% | - |
| 2 | Men with metastatic castration-resistant PCa should have somatic and germline testing for *BRCA1, BRCA2, PALB2 and ATM [carried forward from Round 1]* | 68% | 9% | 23% | - |
| 2 | Men with metastatic castration-resistant PCa should have genetic testing regardless of family history (a multigene panel including at least *BRCA1, BRCA2 and MMR* genes [*MLH1, MSH2, MSH6, PMS2 and EPCAM*]) to inform treatment *[new statement]* | **73%** | 9% | 18% | **Consensus** |
| **3. How would genetic testing results inform PSA testing for prostate cancer?** | | | | | |
| 1 | Men without PCa should have PSA testing discussions if they have *BRCA2* mutation status only | 29% | 6% | 65% | - |
| 1 | Men without PCa should have PSA testing discussions if they have *HOXB13* mutation status only | 29% | 6% | 65% | - |
| 1 | Men without PCa should have PSA testing discussions if they have mutation status in any of the following *BRCA2, BRCA1, HOXB13, ATM, DNA MMR* | **74%** | 9% | 18% | **Consensus** |
| 2 | Men without PCa with a pathogenic variant in a gene known to cause PCa should have PSA testing discussions *[revised based on feedback]* | **100%** | 0% | 0% | **Consensus** |
| 2 | Men without PCa with no detected pathogenic variants and a family history of PCa, particularly if early age at diagnosis, should have PSA testing discussions *[new statement]* | **95%** | 5% | 0% | **Consensus** |
| 2 | Men with localised PCa should have PSA testing discussions if they have a pathogenic variant in *BRCA2,* *BRCA1, HOXB13, ATM, DNA MMR*, (particularly if they have a history of multiple cancers in the same side of the family, early age at diagnosis or Ashkenazi Jewish ancestry) *[new statement]* | 64% | 27% | 9% | - |
| **4. How would genetic testing inform the management of prostate cancer?** | | | | | |
| 1 | Of all genes on multigene panels for non-metastatic PCa, only *BRCA2* should be factored into management discussions (e.g., active surveillance) | 6% | 38% | 56% | - |
| 1 | Of all genes on multigene panels for metastatic PCa, only *BRCA2 and BRCA1* should be factored into management discussions | 12% | 29% | 59% | - |
| 1 | Of all genes on multigene panels for metastatic castration-resistant PCa, only *BRCA2, BRCA1 and ATM* should be factored into management discussions. | 21% | 32% | 47% | - |
| **5. When should genetic counselling be provided?** | | | | | |
| 1 | Before genetic testing to ascertain if the family history warrants genetic testing | **83%** | 14% | 3% | **Consensus*** |
| 1 | Before genetic testing to explain what is going to happen and the possible results, next steps, etc. | **92%** | 8% | 0% | **Consensus*** |
| 1 | After genetic testing for those with a negative result | 50% | 31% | 19% | - |
| 2 | After genetic testing for those with a negative result *[carried forward from Round 1]* | 46% | 33% | 21% | - |
| 1 | After genetic testing for those with a positive result | **94%** | 0% | 6% | **Consensus*** |
| **6. Who should provide genetic counselling?** | | | | | |
| 1 | A specialised genetic counsellor | **78%** | 19% | 3% | **Consensus*** |
| 1 | A GP with accreditation in genetic counselling (e.g., a diploma or a certificate from attending a series of workshops) | 56% | 25% | 19% | - |
| 2 | A GP with accreditation in genetic counselling (e.g., a diploma or a certificate from attending a series of workshops) *[carried forward from Round 1]* | 29% | 38% | 33% | - |
| 2 | A GP with accreditation in genetic counselling (e.g., completion of a short degree with examination and pass mark) *[revised based on feedback]* | 38% | 29% | 33% | - |
| 1 | Allied health professionals or nurses with additional qualifications /accreditation in genetic counselling | **72%** | 19% | 8% | **Consensus*** |
| 1 | A member of the oncology team (mainstream consent pathway) | 50% | 22% | 28% | - |
| 2 | A member of the oncology team (mainstream consent pathway) conducts all counselling *[revised based on feedback]* | 38% | 29% | 33% | - |
| 2 | Partial mainstream consent pathway whereby medical oncologist can order germline testing, provide pre-test counselling and gain consent to test from the patient. Referral to a genetic counsellor or geneticist could then be limited to those patients with a positive test result or no detected abnormality but a familial history that warrants further investigation *[new statement]* | **83%** | 13% | 4% | **Consensus** |
| 1 | A geneticist | 58% | 25% | 17% | - |
| 2 | A geneticist *[carried forward from Round 1]* | 67% | 25% | 8% | - |
| 1 | A psychologist, counsellor or social worker | 14% | 22% | 64% | - |
| 2 | A psychologist, counsellor or social worker *[carried forward from Round 1]* | 0% | 17% | **83%** | **Negative Consensus** |
| **7. How should genetic counselling be carried out?** | | | | | |
| 1 | Psychosocial needs/preferences should dictate mode of counselling | **81%** | 17% | 3% | **Consensus*** |
| 1 | It should be conducted face to face | 33% | 47% | 19% | - |
| 2 | Genetic counselling should be conducted face to face *[carried forward from Round 1]* | 17% | 38% | 46% | - |
| 1 | Genetic counselling can be effectively conducted via videoconferencing/telehealth | **86%** | 14% | 0% | **Consensus*** |
| 1 | It should include comprehensive discussion of the purpose of genetic testing | **92%** | 8% | 0% | **Consensus*** |
| 1 | It should include comprehensive discussion of potential types of results | **94%** | 6% | 0% | **Consensus*** |
| 1 | It should include comprehensive discussion of potential out of pocket costs | **97%** | 3% | 0% | **Consensus*** |
| 1 | It should include comprehensive discussion of cascade/additional family testing | **92%** | 6% | 3% | **Consensus*** |
| 1 | It should include comprehensive discussion of privacy/who has permission to see the results | **94%** | 3% | 3% | **Consensus*** |
| 1 | It should include comprehensive discussion of ramifications on health/income protection insurance | **78%** | 14% | 8% | **Consensus*** |

**Bold** indicates consensus achieved (≥70% agreement).

* indicates consensus achieved in 1st Round of Delphi Panel.

HBOC = hereditary breast and ovarian cancer; PCa = prostate cancer; PSA = Prostate Specific Antigen

**Supplementary Table 2: Level of consensus for genetic testing and genetic counselling among consumers (Round 1 and Round 2)**

| **Round** | **Statements** | **Agree** | **Neutral** | **Disagree** | **Consensus** |
| --- | --- | --- | --- | --- | --- |
| **1. Which men should be considered for genetic testing?** | | | | | |
| 1 | Men with a family history of high-risk hereditary (germline) cancer predisposition genes | **100%** | 0% | 0% | **Consensus*** |
| 1 | Men with family history of multiple cancers on the same side of the family | **96%** | 4% | 0% | **Consensus*** |
| 1 | Men with one or more close blood relatives with a prostate cancer diagnosis at < 60 years | **100%** | 0% | 0% | **Consensus*** |
| 1 | Men with one or more close blood relatives with metastatic (advanced) prostate cancer | **100%** | 0% | 0% | **Consensus*** |
| 1 | Men with one or more close blood relatives who died of prostate cancer | **96%** | 4% | 0% | **Consensus*** |
| 1 | Men with prostate cancer and a family history of Hereditary Breast and Ovarian Cancer (HBOC) syndrome (including breast, ovarian, pancreatic, prostate) | **89%** | 11% | 0% | **Consensus*** |
| 1 | Men with prostate cancer and a family history of prostate cancer | **96%** | 4% | 0% | **Consensus*** |
| 1 | Men with prostate cancer and a family history of Lynch Syndrome (colorectal, upper gastrointestinal tract, endometrial, ovarian, pancreatic prostate, upper urinary tract cancers and sebaceous adenocarcinomas) | **81%** | 19% | 0% | **Consensus*** |
| 1 | Men with non-metastatic prostate cancer (cancer that has not spread outside the prostate gland) and Ashkenazi Jewish ancestry | **70%** | 22% | 7% | **Consensus*** |
| 1 | Men with non-metastatic prostate cancer and Grade Group 4 (Gleason score 8 when cells look abnormal and may grow at a moderate to fast rate) or above | **85%** | 7% | 7% | **Consensus*** |
| 1 | Men with non-metastatic prostate cancer and advanced disease (T3 - cancer that has spread outside the prostate gland, but not progressed to other parts of the body) | **85%** | 4% | 11% | **Consensus*** |
| 1 | Men with non-metastatic prostate cancer and intraductal/ductal pathology (aggressive cancer found in the prostatic ducts) | **81%** | 11% | 7% | **Consensus*** |
| 1 | All men with metastatic prostate cancer should have somatic genetic testing (testing for acquired [not inherited] mutations in the tumour tissue) | **89%** | 11% | 0% | **Consensus*** |
| 1 | All men with metastatic prostate cancer should have germline genetic testing (testing for inherited genetic mutations via cheek swab, spit or blood sample) | **100%** | 0% | 0% | **Consensus*** |
| 1 | Men with metastatic prostate cancer and somatic genetic testing results showing mutation in cancer risk genes should have germline genetic testing (factoring in personal and family history) | **100%** | 0% | 0% | **Consensus*** |
| **2. When should genetic counselling be provided?** | | | | | |
| 1 | Before genetic testing to ascertain if the family history warrants genetic testing | **93%** | 7% | 0% | **Consensus*** |
| 1 | Before genetic testing to explain what is going to happen and the possible results, next steps, etc. | **96%** | 4% | 0% | **Consensus*** |
| 1 | After genetic testing for those with a negative result | **74%** | 19% | 7% | **Consensus*** |
| 1 | After genetic testing for those with a positive result | **96%** | 4% | 0% | **Consensus*** |
| **3. Who should provide genetic counselling?** | | | | | |
| 1 | A specialised genetic counsellor | **89%** | 11% | 0% | **Consensus*** |
| 1 | A GP with accreditation in genetic counselling (e.g., a diploma or a certificate from attending a series of workshops) | **85%** | 15% | 0% | **Consensus*** |
| 1 | Allied health professionals or nurses with additional qualifications /accreditation in genetic counselling | **81%** | 19% | 0% | **Consensus*** |
| 1 | A member of the oncology team (mainstream consent pathway) | 67% | 26% | 7% | - |
| 2 | A member of the oncology team (mainstream consent pathway) conducts all counselling *[revised based on feedback]* | **76%** | 14% | 10% | **Consensus*** |
| 2 | Partial mainstream consent pathway whereby medical oncologist can order germline testing, provide pre-test counselling and gain consent to test from the patient. Referral to a genetic counsellor or geneticist could then be limited to those patients with a positive test result or no detected abnormality but a familial history that warrants further investigation *[new statement]* | **90%** | 5% | 5% | **Consensus** |
| 1 | A geneticist | **70%** | 22% | 7% | **Consensus*** |
| 1 | A psychologist, counsellor or social worker | 41% | 41% | 19% | - |
| 2 | A psychologist, counsellor or social worker | 43% | 29% | 29% | - |
| **4. How should genetic counselling be carried out?** | | | | | |
| 1 | Psychosocial needs/preferences should dictate mode of counselling | **74%** | 22% | 4% | **Consensus*** |
| 1 | It should be conducted face to face | **85%** | 15% | 0% | **Consensus*** |
| 1 | It can be effectively conducted via videoconferencing/telehealth | 52% | 37% | 11% | **-** |
| 2 | It can be effectively conducted via videoconferencing/telehealth *[carried forward from Round 1]* | 52% | 43% | 5% | **-** |
| 1 | It should include comprehensive discussion of the purpose of genetic testing | **85%** | 15% | 0% | **Consensus*** |
| 1 | It should include comprehensive discussion of potential types of results | **89%** | 11% | 0% | **Consensus*** |
| 1 | It should include comprehensive discussion of potential out of pocket costs | **93%** | 7% | 0% | **Consensus*** |
| 1 | It should include comprehensive discussion of cascade/additional family testing | **93%** | 7% | 0% | **Consensus*** |
| 1 | It should include comprehensive discussion of privacy/who has permission to see the results | **89%** | 11% | 0% | **Consensus*** |
| 1 | It should include comprehensive discussion of ramifications on health/income protection insurance | **96%** | 4% | 0% | **Consensus*** |

**Bold** indicates consensus achieved (≥70% agreement)

* indicates consensus achieved in 1st Round of Delphi Panel.
